# Reply to Chen, A.; Zhang, X. Comment on “Golčić et al. Evaluation of Systemic Treatment Options for Gastrointestinal Stromal Tumours. *Cancers* 2023, *15*, 4081”

**DOI:** 10.3390/cancers15235619

**Published:** 2023-11-28

**Authors:** Marin Golčić, Robin L. Jones, Paul Huang, Andrea Napolitano

**Affiliations:** 1 Department of Radiotherapy and Oncology, Clinical Hospital Center Rijeka, Krešimirova 42, 51000 Rijeka, Croatia; 2 Sarcoma Unit, The Royal Marsden NHS Foundation Trust, Fulham Road, London SW3 6JJ, UK; robin.jones4@nhs.net (R.L.J.); andrea.napolitano@rmh.nhs.uk (A.N.); 3 Division of Molecular Pathology, The Institute of Cancer Research, Sutton SM2 5NG, UK; paul.huang@icr.ac.uk

We appreciate the comment made by Chen et al. on our manuscript evaluating the systemic treatment options for gastrointestinal stromal tumours (GIST) [1]. Their comment serves as a reminder of the complexity and issues surrounding the treatment of GIST, and we are thankful to the authors for carefully reading our manuscript.

Firstly, we agree with Chen et al. [2] that the European Society for Medical Oncology (ESMO) Guidelines state sunitinib is the standard second-line treatment for patients with advanced GIST [3]. However, the choice of the second-line treatment option depends on the GIST mutational status and the availability of the drug. For example, for patients with GIST with the *PDGFRA D842V* mutation progressing on avapritinib, sunitinib should not be used as a standard second-line therapy due to inefficiency [4,5]. Patients with GIST harbouring *KIT* exon mutations 11 demonstrated worse progression-free survival compared to patients with *KIT* exon 9 [6], which could also play a role in deciding the subsequent lines of therapy. Furthermore, Chefchaouni et al. demonstrated the profound impact of anti-cancer drug shortages in Morocco, including sunitinib [7]. Hence, having several treatment options upon progression to imatinib 400 mg daily dose is valuable in the real-world setting, as imatinib dose escalation can result in a measurable clinical benefit, albeit at the cost of a higher percentage of grade 3–5 toxicities [8].

Secondly, we concur with Chen et al. [2] that the table titled “Systemic Therapy Agents and Regimens for Unresectable, Progressive, or Metastatic Disease” in the National Comprehensive Cancer Network (NCCN) Guidelines does not explicitly mention imatinib dose elevation in the second-line treatment [4]. However, we point Chen et al. towards the treatment algorithm in the same guidelines (GIST-4 and GIST-5), which clearly state that the dose escalation of imatinib is an option upon progression on imatinib 400 mg, along with a switch to alternative tyrosine kinase inhibitors (TKI).

Thirdly, the authors comment that the dose escalation of imatinib does not fall under the category of second-line treatment [2]. However, we disagree with such a statement due to several reasons. As mentioned, the NCCN Guideline’s treatment algorithm considers TKI a standard first-line therapy, while upon progression, a “Follow-up therapy” (which we would define as second-, third-, fourth-line, etc.) is recommended, which includes dose escalation of imatinib [4]. While the ESMO Guidelines do not explicitly define imatinib escalation as second-line therapy in the text, the treatment algorithm for advanced or metastatic GIST (Figure 1 in the same manuscript) clearly demonstrates the distinction between the two doses and points to the imatinib dose escalation as a potential option upon progression to the lower dose [3]. The categorisation of imatinib 800 mg as a second-line treatment is mentioned in various research [9,10,11], including the manuscript co-authored by the writers of the ESMO Guidelines [3], which states that “… *2nd-line treatment with either imatinib 800 mg/day or sunitinib may be considered as subsequent treatment..*.” [12]. The rechallenge study from the same team also included patients who “*…had received imatinib (800 mg) as a second-line therapy*” [13]. Finally, despite the limited success of the therapy, not recognising imatinib dose escalation as a clear second-line therapy would complicate any clinical trial challenging imatinib in the first-line and would give a disadvantage to any tested medication.

We acknowledge that the treatment of patients with advanced GIST, which could combine localised therapy, systemic therapy, and inclusion in clinical trials, can blur the distinction between the lines of therapy. The categorization of the lines of therapy primarily serves for learning and understanding purposes, and each patient with GIST should have an individualised treatment plan made by a multidisciplinary team in the centre with experience treating sarcoma.

## References

[1] Golčić M., Jones R.L., Huang P., Napolitano A. (2023). Evaluation of Systemic Treatment Options for Gastrointestinal Stromal Tumours. Cancers.

[2] Chen A., Zhang X. (2023). Comment on Golčić et al. Evaluation of Systemic Treatment Options for Gastrointestinal Stromal Tumours. *Cancers* 2023, *15*, 4081. Cancers.

[3] Casali P.G., Blay J.Y., Abecassis N., Bajpai J., Bauer S., Biagini R., Bielack S., Bonvalot S., Boukovinas I., Bovee J.V.M.G. (2022). Gastrointestinal stromal tumours: Esmo-euracan-genturis Clinical Practice Guidelines for diagnosis, treatment and follow-up. Ann. Oncol..

[4] National Comprehensive Cancer Network. Gastrointestinal Stromal Tumors (Version 1.2023). http://www.nccn.org/professionals/physician_gls/pdf/gist.pdf.

[5] Sun Y., Yue L., Xu P., Hu W. (2022). An overview of agents and treatments for PDGFRA-mutated gastrointestinal stromal tumors. Front. Oncol..

[6] Demetri G.D., van Oosterom A.T., Garrett C.R., Blackstein M.E., Shah M.H., Verweij J., McArthur G., Judson I.R., Heinrich M.C., Morgan J.A. (2006). Efficacy and safety of sunitinib in patients with advanced gastrointestinal stromal tumour after failure of imatinib: A randomised controlled trial. Lancet.

[7] Cherif Chefchaouni A., Moutaouakkil Y., Adouani B., Tadlaoui Y., Lamsaouri J., Bousliman Y. (2022). Impact of anti-cancer drugs shortages in oncology and hematology departments in a Moroccan hospital. J. Oncol. Pharm. Pract..

[8] Blanke C.D., Rankin C., Demetri G.D., Ryan C.W., von Mehren M., Benjamin R.S., Raymond A.K., Bramwell V.H., Baker L.H., Maki R.G. (2008). Phase III randomized, intergroup trial assessing imatinib mesylate at two dose levels in patients with unresectable or metastatic gastrointestinal stromal tumors expressing the kit receptor tyrosine kinase: S0033. J. Clin. Oncol..

[9] Zhang X., Liu X., Wu X.J., Pan Z., Lu H.S., He Y. (2017). Imatinib dose escalation versus sunitinib as a second-line treatment against advanced gastrointestinal stromal tumors: A nationwide population-based cohort study. Oncotarget.

[10] Mohammadi M., Jansen-Werkhoven T.M., Ijzerman N.S., den Hollander D., Bleckman R.F., Oosten A.W., Desar I.M., Reyners A.K., Steeghs N., Gelderblom H. (2022). Dutch Gastrointestinal Stromal Tumor (GIST) Registry Data Comparing Sunitinib with Imatinib Dose Escalation in Second-Line Advanced Non-KIT Exon 9 Mutated GIST Patients. Target Oncol..

[11] Huang S., Liu X., Guo X., Wu H., Lu H., Pan Z., Cai S., Wu X., Zhang X. (2023). Sunitinib versus imatinib dose escalation after failure of imatinib standard dose in patients with advanced Gastrointestinal stromal tumors—A real-world multi-center study. Transl. Oncol..

[12] Vincenzi B., Nannini M., Fumagalli E., Bronte G., Frezza A.M., De Lisi D., Ceruso M.S., Santini D., Badalamenti G., Pantaleo M.A. (2016). Imatinib dose escalation versus sunitinib as a second line treatment in KIT exon 11 mutated GIST: A retrospective analysis. Oncotarget.

[13] Vincenzi B., Nannini M., Badalamenti G., Grignani G., Fumagalli E., Gasperoni S., D’Ambrosio L., Incorvaia L., Stellato M., Spalato Ceruso M. (2018). Imatinib rechallenge in patients with advanced gastrointestinal stromal tumors following progression with imatinib, sunitinib and regorafenib. Ther. Adv. Med. Oncol..

